# Changes in BDNF Concentration in Men after Foam Roller Massage

**DOI:** 10.3390/cells13181564

**Published:** 2024-09-17

**Authors:** Eugenia Murawska-Ciałowicz, Maria Ciałowicz, Adam Rosłanowski, Agnieszka Kaczmarek, Katarzyna Ratajczak-Wielgomas, Alicja Kmiecik, Aleksandra Partyńska, Piotr Dzięgiel, Waldemar Andrzejewski

**Affiliations:** 1 Physiology and Biochemistry Department, Wroclaw University of Health and Sport Sciences, 51-612 Wroclaw, Poland; agnieszka.kaczmarek@awf.wroc.pl; 2 Physiotherapy Faculty, Wroclaw University of Health and Sport Sciences, 51-612 Wroclaw, Polandpiotr.dziegiel@umw.edu.pl (P.D.); waldemar.andrzejewski@awf.wroc.pl (W.A.); 3 Division of Histology and Embryology, Department of Human Morphology and Embryology, Wroclaw Medical University, 50-368 Wroclaw, Poland; katarzyna.ratajczak-wielgomas@umw.edu.pl (K.R.-W.); alicja.kmiecik@umw.edu.pl (A.K.); aleksandra.partynska@umw.edu.pl (A.P.)

**Keywords:** foam rollers, massage, BDNF

## Abstract

Massage is one of the oldest forms of therapy practiced since ancient times. Nowadays, it is used in sports practice, recovery from injury, or supportive therapy for various conditions. The practice of massage uses a variety of instruments that facilitate massaging while relieving the stress on the masseur. One of them is a foam roller. Although roller massage is widely used, there are still no scientific studies describing the biological mechanisms of its effects on the body. The purpose of our study was to analyze the effect of roller massage on BDNF levels in men undergoing self-massage 4x/week/7 weeks. The control group consisted of men who did not perform self-massage. Before the test and after the first, third, fifth, and seventh weeks of self-massage, the study participants’ blood was drawn, the serum BDNF was determined, and the results were subjected to analysis of variance by ANOVA test. After the first week of self-massage, an increase in BDNF concentration was observed in the self-massage group compared to the control group (*p* = 0.023). Similarly, changes were observed in week five (*p* = 0.044) and week seven (*p* = 0.046). In the massaged group, BDNF concentrations were significantly higher after the first week of self-massage compared to baseline. In the third week of the study, BDNF decreased to a value comparable to the baseline study, then increased significantly in the fifth and seventh weeks compared to the value recorded in the third week (*p* = 0.049 and *p* = 0.029). It was significantly higher in week seven compared to week five (*p* = 0.03). Higher concentrations of BDNF in subjects undergoing roller self-massage may be one of the biological mechanisms justifying the therapeutic effects of massage in both sports and clinical practice. Studies analyzing the stimulation of BDNF synthesis through various massage techniques should be performed on a larger group of healthy individuals, patients after trauma of multiple origins, and sick people with indications for therapeutic massage.

## 1. Introduction

Massage is one of the oldest forms of therapy, known and practiced in many ancient cultures, including the Greeks and Romans, China, Japan, Mesopotamia, Egypt, and Korea [1], based on the availability of the form and awareness of its beneficial effects on the body.

Due to its health-promoting effects and availability, massage is very popular in modern times and is used in the treatment of many diseases. It is widely applied in sports practices, in post-training and post-injury recovery, as well as in the area of wellness and fitness [2].

Massage involves the use of various strengths and forms of touch, from gentle stroking through pressure and rubbing to firm kneading. Various structures and receptors located in skin, connective tissue, muscle, tendon, bone, and internal organs are activated through the application of a mechanical stimulus. The mechanical action of massage promotes the occurrence of beneficial local and systemic changes.

Some of the most important therapeutic effects of massage include pain relief, reduction in muscle tension, increased flexibility, improved blood supply to the massaged tissues, and an increase in their temperature. Better-perfused skin and muscles allow for faster exercise results [3] and tissue regeneration after injury, e.g., after muscle and tendon damage following sports injuries [2]. Massage regulates blood pressure, reduces stress and emotional tension, improves mood (it is used in treating depression), has immunomodulatory effects [4], and alleviates the effects of the inflammatory process [5,6].

All the observed effects of massage are the result of the activation of multiple biological mechanisms from the molecular level to changes in tissue structure and function [7].

Massage induces the phenomenon of mechanotransduction, defined as the conversion of a mechanical stimulus or energy into a chemical signal triggering an intracellular signal cascade. This promotes protein synthesis due to the secretion of IGF-1 or testosterone and hypertrophy of skeletal muscle fibers [8]. It also stimulates the synthesis of vascular endothelial growth factor (VEGF), the main factor that stimulates angiogenesis [9], and fibroblast growth factor (FGF) secreted by tendon tissue structures, provoking structural changes in collagen fibers [10]. This may be relevant for scar regeneration and reparation after injury [11].

In addition to those mentioned, one of the beneficial effects of massage is the stimulation of nerve transmission and the synthesis of chemicals with different effects (such as endorphins, serotonin, dopamine, oxytocin, or the endocannabinoid system), which promote improved mood [12,13]. Part of the endocannabinoid system are arachidonyloetanoloamide (anandamide) and 2-arachidonyloglicerol (2-AG). Both of them are stimulated during physical exercise and are responsible for the “runner’s high” phenomenon known in sports [14].

Brain-derived neurotrophic factor (BDNF), considered one of the most potent factors in neural plasticity, is involved in nerve transmission and nerve fiber regeneration processes. It stimulates neurogenesis, dendritogenesis, or synaptogenesis. It is responsible for the growth, differentiation, maturation, and survival of neurons, as well as the maturation of dendritic spines. It is involved in long-term potentiation (LTP) induction processes, and it regulates synaptic plasticity [15]. It has a regulatory effect on inflammatory processes. BDNF has a beneficial effect on the regulation of the hypothalamic-pituitary-adrenal (HPA) axis, participates in muscle hypertrophy, learning and memory processes, mood improvement, and has antidepressant and anti-inflammatory effects [16,17,18]. Its highest concentration is observed in the brain, and one of the factors that stimulate BDNF secretion in the brain is skeletal muscle contraction during exercise. BDNF belongs to the myokines and neurokines.It exerts its biological effects through a specific receptor, which is tyrosine kinase B (TrkB). Activation of this receptor activates protective and anti-apoptotic effects [19,20]. The highest expression of the BDNF receptor is particularly observed in hippocampal neurons [21].

Roosterman et al. [22] also postulate that mammalian skin, as a neuroimmunoendocrine organ, is the site of production of many neurotrophic growth factors, including BDNF and its receptor TrkB. Growth factors in the skin are essential for growth, proliferation, and maintenance of information flow. Cutaneous neurotrophins are expressed in sensory and sympathetic neurons and non-neuronal cells [23,24], thus regulating various biological processes, such as nociception, proprioception, mechanoreception, nerve growth, development, apoptosis, epidermal homeostasis, inflammation, hair growth, and melanogenesis. Neurotrophins participate in the skin’s neuroimmune network.

Because markedly increased secretion of BDNF is observed in skeletal muscle during contraction, massage (mechanotransduction) and exercise appear to be the main physical factors stimulating its expression. In addition, it was found that mechanical irritation of the skin can affect BDNF secretion.

Given the numerous beneficial effects of massage, including participation in regenerative processes, BDNF may be one of the important biological mechanisms contributing to the innervation of massaged tissues and influencing processes in many organs, including muscles, nerve fibers, and the brain.

Despite the growing knowledge of massage’s effects on the body and molecular mechanisms, its effects are not fully understood. Only a few authors have investigated whether BDNF is expressed or secreted following a massage, including roller massage, despite the growing interest and use of this device in sports physiotherapy, rehabilitation, and wellness [25].

Despite experimental studies on the role of BDNF in cutaneous neural transmission, to the best of our knowledge, the only existing scientific reports in this area are those by Wu et al. [26], Ribeiro et al. [27], and Shi et al. [28]. Wu et al. [26] observed an increase in BDNF levels after a 4-week aromatherapy massage. Ribeiro et al. [27] conducted a study in patients with fibromyalgia in which, after vibration training, an increase in BDNF levels was observed. The study on a rat model of stroke reported decreased miR-206 expression and increased BDNF levels after meridian massage [28].

Since the effect of massage on BDNF expression is not elucidated, the purpose of our study was to analyze changes in the concentration of this protein in the blood of men subjected to 8 weeks of self-massage sessions using foam rollers.

## 2. Materials and Methods

### 2.1. Study Group

This study involved healthy men between the ages of 19 and 25 who were students in the first year of the General Tadeusz Kościuszko Military University of Land Forces in Wroclaw, Poland. They were divided into two groups: those subjected to roller massage (M), *n* = 12, and a control group not subjected to the massage (C), *n* = 8. The subjects were assigned to the groups randomly. Both groups of subjects followed the same daily schedule, participated in military training, and remained on the same diet. Exclusion criteria from the study were acute and chronic diseases, surgery, mechanical injuries to the musculoskeletal system, inflammation, elevated body temperature, infections, including infections localized to the massage site, smoking, cancer, diabetes, osteoporosis, aneurysm, and skin lesions. The design of the experiment received a favorable opinion from the Research Ethics Committee at the Academy of Physical Education in Wroclaw No. 32/18 (10.10.2018).

### 2.2. Methods

#### 2.2.1. Anthropological Characteristics

The subjects’ weight and height, body composition, and body mass index (BMI) were measured to assess the anthropological profile. A TANITA type BC-418 MA device was used to assess body composition, with which total (kg) and percentage (%) of body fat (FAT), fat-free mass (kg), muscle mass (kg), percentage of body water (BW%), and bone mass (kg) were measured. Basal metabolism rate (BMR) was calculated. No statistically significant differences were found between the groups. The basic anthropological data of the study participants are presented in Table 1.

#### 2.2.2. Foam Roller Massage

The subjects in the massage group (M) performed self-massage using a hard roller (length 33 cm × diameter 14 cm). The roller had an irregular carbon surface. It was made of EVA foam from Coolmed (Myslenice, Poland).

**Figure 1:**
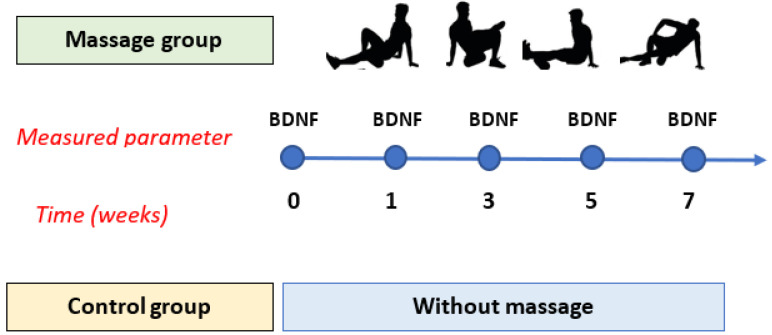
Scheme of collecting blood for testing.

The procedure for performing the massage was as follows: (1) massage of the posterior side of the lower leg (gastrocnemius muscle); (2) massage of the posterior side of the thigh (biceps femoris muscle, semitendinosus muscle, semimembranosus muscle); (3) massage of the medial part of the thigh (adductor longus muscle, brevis, and Magnus); (4) massage of the lateral part of the thigh (quadriceps muscle); (5) massage of the gluteal area (gluteus maximus muscle); (6) massage of the anterior side of the thigh (quadriceps muscle). The rolling of each muscle group lasted 1.5 min at a speed of 2.5 cm/sec and body weight pressure. Each limb was massaged separately for 9 min. The total massage time for both limbs was 18 min. It was always performed around noon, 4 times a week for 7 weeks. Roller training was conducted by professional physiotherapists. The participants performed all exercises according to the physiotherapist’s instructions to maintain the appropriate order of exercises, their duration, and the rhythm of the exercises (Figure 1).

### 2.3. Biochemical Tests

Fasting 5 mL of blood was collected from the ulnar vein into an S-Monovette tube (Sarstedt, Nümbrecht, Germany). The tubes were left at 20–23 °C until clot formation (10–15 min). After this time, the blood was centrifuged for 10 min (2000× *g*) at room temperature. After centrifugation, serum was extracted from the tube, divided into several 1.5 mL portions, and frozen at −80 °C until the analyses were made. Fasting blood was collected at baseline (week 0), after the 1st week of self-massage (week 1), after the third (week 3), after the fifth (week 5), and after the seventh week of the study (week 7). After all samples were collected, the serum was thawed, and BDNF concentration was determined by ELISA using Abcam’s reagent kit (ab212166—Human BDNF SimpleStep ELISA^®^ Kit, Cambridge, UK). The coefficient of variation (CV) intra-assay was CV = 2.8%, and inter-assay CV = 5.3%. The minimum detectable dose in this method was 2.4 pg/mL. Each sample was assayed twice. An average value was drawn from both measurements. The results obtained were subjected to statistical analysis.

### 2.4. Statistical Analysis

Statistical analysis of the obtained results and the minimal group size were performed using Statistica 13.1 (USA). For the purpose of BDNF analysis, the minimal number of 12 participants was calculated before the experiment. The distribution of the results was calculated using the Shapiro–Wilk test, and the homogeneity of variance was examined using the Leven test. To verify whether massage had a statistically significant effect on BDNF concentrations, a one-way analysis of variance with Fisher’s post hoc NIR test was used. The percentage of change in BDNF concentration during the following weeks of training with roller self-massage compared to the initial value was calculated according to the formula: % change = 100 × [(final value − initial value)/initial value] [29].

## 3. Results

### 3.1. Anthropological Parameters

Table 1 shows the results of the anthropological parameters of the two groups studied. There were no statistically significant differences in anthropological parameters between the groups before and after the seven-week study.

### 3.2. BDNF Levels

**Figure 2:**
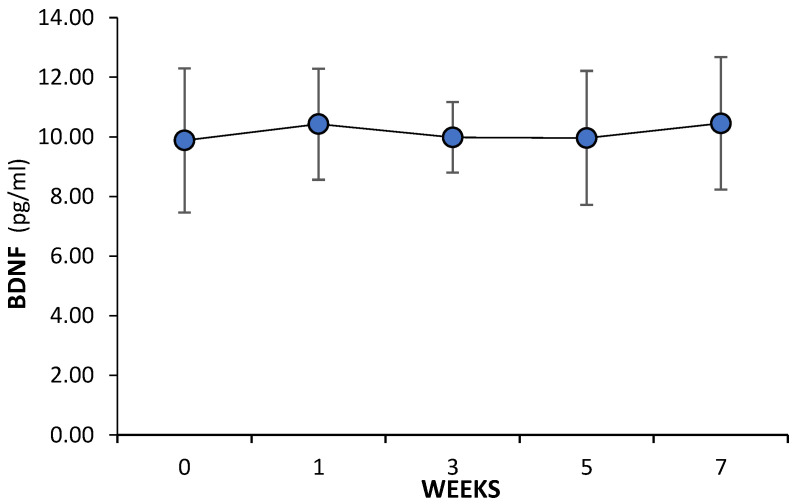
BDNF level in the C group in the subsequent weeks of the study.

**Figure 3:**
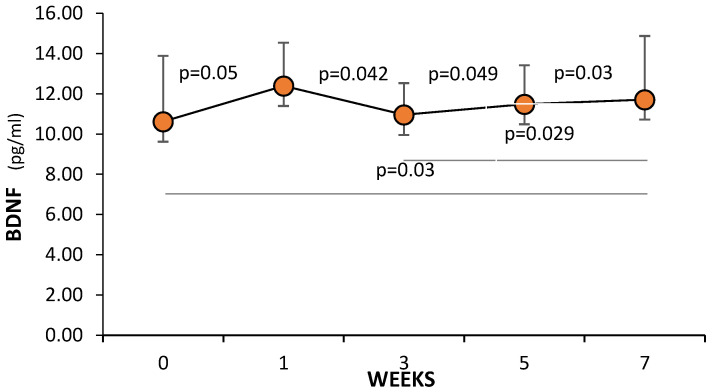
BDNF level in the M group in the subsequent weeks of the study.

During the seven weeks of the study, BDNF levels in the control group did not change (Figure 2), while in the study group, BDNF increased compared to the initial value (week 0) (Figure 3). BDNF concentration at week 7 of the study was 11.75% (*p* = 0.03) higher than the value recorded in week 0. An increase in BDNF concentration was observed after the first week, when its value was significantly higher by 12.22% compared to the initial value (*p* = 0.05). In week 3, BDNF levels were significantly reduced by 11.65% (*p* = 0.042), compared to the value recorded in week 1, with no statistically significant differences from baseline. In week 5, BDNF concentrations increased from the value recorded in week 3 by 12.7% (*p* = 0.049). In week 7, further increases in BDNF concentrations were recorded compared to the value recorded in week 5. This was an increase of another 7.1% (*p* = 0.03). Between BDNF concentrations in weeks 3 and 7, statistically significant differences were noted (*p* = 0.029).

**Figure 4:**
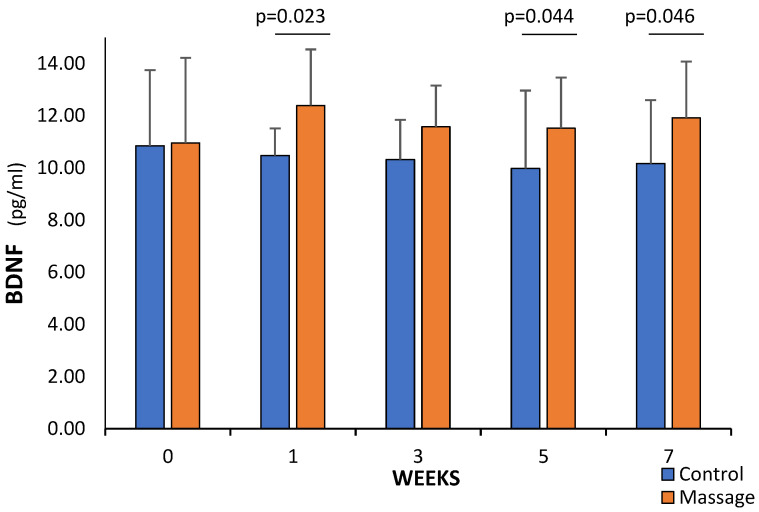
Comparison of BDNF level between the C and M groups in the subsequent weeks of the study.

Statistically significant differences were found between the C and M groups in each of the weeks studied, except between weeks 0 and 3 (Figure 4). In week 1, BDNF was 15.7% higher in the massage group (*p* = 0.023), 20.7% higher in week 5 (*p* = 0.044), and 16.6% higher in week 7 (*p* = 0.046) (Figure 4).

## 4. Discussion

We observed that roller self-massage for seven weeks resulted in an increase in serum BDNF levels in young, healthy men. A significant increase in BDNF relative to baseline was found after the first week of the study. At each of the observed weeks except baseline and week three, BDNF levels in the massaged group were also significantly higher compared to the control group. This may indicate that mechanical irritation of the skin, fascial system, and muscles during massage stimulates biological mechanisms leading to stimulation of BDNF synthesis. In our study, lower BDNF levels in week 3 reported for the massage group could be explained by the influence of obligatory military training conducted during our research and the physical overload of soldiers after additional training. Together with foam roller massage, it could result in a decrease in BDNF concentration. We can speculate in this way because a lower but not significant value of BDNF was observed also in the control group at this time point.

The deformation of connective tissue, a component of various anatomical structures, such as fascia, tendons, ligaments, skin, muscles, or scaffolding for internal organs, causes elastic deformation of collagen fibers. As a result, the fibers are stressed, and mechanical forces are transmitted deep into the tissues, into the cells on the filaments of the cytoskeleton, and into the cell nuclei [30,31,32,33,34,35]. The transfer of mechanical energy into the cell is mediated by cell adhesion proteins (integrins, selectins, cadherins) with transmembrane receptor structures, as well as stretch-activated ion channels and other receptors that change their conformation under the influence of a stress/mechanical stimulus [36]. Adhesion proteins participate in cell migration during development and repair. They mediate bidirectional signaling between the extracellular matrix and cytoskeleton across the cell membrane, activate signal transduction pathways, and mediate the activation of cellular signals. They are also involved in the organization of the intracellular cytoskeleton and the movement of new receptors to the cell membrane [37]. Stimulation of adhesion proteins or ion channels by a strain or mechanical stimulus induces activation of molecular mechanisms in the cell, such as G-protein activation, induction of secondary messengers, activation of the RPTK, JAK/STAT kinase cascade, or activation of PI3K, which leads to several physiological phenomena. This includes stimulation of IGF-1 synthesis and muscle hypertrophy through activation of the mTOR pathway [8,38,39,40,41,42]. Other extracellular matrix proteins are also involved in integrin-mediated regeneration, including, in addition to collagen, fibronectin, laminin, or tenascin, described in more detail in a study by Nieuwenhuis et al. [43]. Activation of integrins by a mechanical stimulus accelerates trophic processes and promotes improved blood supply and innervation of damaged tissues [2].

According to Shafrir and Fargacs [30], mechanotransduction through the cytoskeleton is no longer a surprising phenomenon today. According to the authors, all cells in the body are exposed to external forces and sense these forces. What is surprising, however, is that physical factors control biological processes, such as cell differentiation, proliferation, gene expression, and especially signal transduction [44,45].

Based on the results obtained in the present study, it can be assumed that the increase in BDNF concentration stimulated by roller massage is also an effect of mechanotransduction. Massage dosed systematically over an extended period promotes the reorganization of the structure of the massaged muscles and collagen structures. Reorganization of the structure of myocytes and repair of damaged collagen fibers is related to stimulation of angiogenesis and improvement of vascularization of the regenerated area, as well as regeneration of nerve fibers. In his study, Roslanowski et al. [11] observed increased levels of VEGF and FGF after roller massage.

We agree that the increase in BDNF levels in the blood of massagers is a result of the mechanical factor of pressure on the skin, fascia, and muscles, as well as the receptors within their structure. Crane et al. [46] observed an increase in focal adhesion kinase (FAK) activity of extracellular signal-regulated kinases 1 and 2 (ERK1/2) and PGC- several hours after massage as well as activation of its COX7B and ND1 pathways. PGC-1α (peroxisome proliferator-activated receptor γ coactivator 1α) enhanced mitochondrial biogenesis through irisin-mediated stimulation of BDNF expression.

According to Cefis et al. [47], BDNF produced by skeletal muscles during exercise comes from endothelial cells and is stimulated by nitric oxide (NO). Osol et al. [48] believe that mechanotransduction in vascular smooth muscle, understood as a cellular response (contraction, secretion, growth, division), is transmitted by increasing pressure or wall stretch.

It is also interesting to note that the presence of BDNF and the TrkB receptor was found in different skin cell populations, such as sensory receptors, endothelial cells, fibroblasts, melanocytes, and keratinocytes [49], where they participate in various responses, including neurohormonal responses [50]. In situ, expression of BDNF was observed in nerve fibers of the skin and myocytes of extensor and subcutaneous muscle [49], which only confirms our earlier conclusion.

According to Slominski et al. [50], various cells of the epidermis and skin, together with keratinocyte and melanocyte receptors, as well as Langerhans and Markel cells, sensory nerves, and an extensive network of dendrites, create their own neuroimmunoendocrine communication network to ensure the maintenance of proper homeostasis. In fact, the skin has been recognized as the “brain on the outside” [51] and the organ of stress. By creating a close connection with the central stress axes, it contributes to maintaining the homeostasis of the entire organism [52].

Moreover, the skin, as a neuroendocrine organ, is able to secrete many different biologically active substances that are able to take part in communication with other organs. One of them is the glucagon-like peptide-1 receptor (GLP-1R). This receptor plays a pivotal role in glucose homeostasis [53]. GLP-1R is detected in the pancreas, liver, lung, kidney, heart, brain, intestine spleen, thymus, and lymph nodes of mice [54]. The presence of GLP-1Rs was also reported in mouse keratinocytes [55] as well as in healthy human keratinocytes and in patients with psoriasis in immune cells [53].

It has also been found that GLP-1R signaling contributes to the regulation of the proliferation of both thymocytes and peripheral T cells. The discovery of the presence of GLP-1R in skin cells may expand knowledge about the role in the functioning of the immune system and be the basis for explaining the beneficial, regulatory effect of massage on the body, including increasing the immune response.

A study by Carroll et al. [56] conducted on a mouse model shows that BDNF is a survival factor for some sensory neurons during development. Cutaneous slow-adapting mechanoreceptors (SAMs), responsible for touch and pressure sensation, require BDNF in postnatal life for the course of normal mechanotransduction. Carroll et al. [56] observed that neurons lacking BDNF showed a significant reduction in mechanical sensitivity. The authors believe that the involvement of BDNF in sensing mechanical stimuli is an important component of survival through SAM regulation. Mice that lacked nerve growth factor or its receptor could show severe loss of nociceptors. SAM neurons, which are required for accurate tactile discrimination, show severely impaired mechanical sensitivity with reduced BDNF levels. Thus, it can be hypothesized that the increase in BDNF levels observed in our study in the self-massage group may be of dermal (various types of skin cells), neural (sensory receptors of skin, fascia, and muscle), muscular (muscle fibers), and vascular (endothelial cells) origin.

There is more and more evidence supporting the claim that massage can have anabolic effects and that it promotes increasing muscle mass, reducing muscle loss [57], and increasing muscle strength and size [58]. There is a strong possibility that mechanical factors converted into intracellular or biochemical signals are important in the process of repair, regeneration, or growth and promote pro-hypertrophic signals [5,59]. Regeneration of damaged tissues promotes muscle hypertrophy as a result of IGF-1 or testosterone secretion [8]. The significant effects of IGF-1 on the nervous system and nerve transmission, including effects on BDNF synthesis, are described extensively by Arjunana et al. [60].

As a result of the activation of regenerative or repair processes, satellite cells are involved in the process of muscle hypertrophy, the stimulation of which promotes the synthesis of new myofibrillar and sarcoplasmic proteins [58]. Wei-feng and Ru-bao [61] reported that during skeletal muscle regeneration, the Wnt/β-catenin signaling pathway plays an important role in regulating myoblast differentiation. Massage is thought to delay skeletal muscle atrophy, probably by regulating satellite cell proliferation and differentiation through the Wnt/β-catenin signaling pathway.

Some authors demonstrated that muscle BDNF participates in the cellular regeneration phase [62,63]. Clow and Jasmin [63] studied skeletal muscle BDNF-depleted mice and BDNF-/- mice and showed that BDNF positively regulates satellite cell function during muscle regeneration. A study by Mousavi et al. [62] shows that BDNF expression correlates with the expression of Pax3, a marker of muscle progenitor cells. In addition, it was observed that BDNF was expressed in Pax7+ satellite cells and that high levels of BDNF were found in myoblasts. According to the authors, BDNF plays an important regulatory function during myogenic differentiation and plays a key role in maintaining muscle progenitor cell populations.

Massage-induced muscle tension can not only activate pro-hypertrophic signaling pathways but can also reduce exercise-induced damage and inflammation. Massage is a powerful stimulus used to speed up muscle recovery after training and promote muscle growth. The regeneration of damaged muscle fibers or collagen structures is accompanied by the formation of new nerve connections or the regeneration of damaged neurons [43]. BDNF is undoubtedly involved in this process, as it is one of the most potent neurokinins stimulating neurogenesis, synaptogenesis, and dendritogenesis. Our research shows that BDNF synthesis is also stimulated in healthy tissues, and massage is a factor in promoting better tissue innervation.

The results of our research may carry an important social message. Knowledge of the therapeutic effect of massage can be used in the prevention and treatment of conditions leading to muscular atrophy and denervation of muscle tissue.

According to various studies [61,64,65], many post-trauma patients, or those suffering from conditions known as post-traumatic stress disorder (PTSD), suffer from peripheral nerve damage, resulting in disability and impaired quality of life. Sensory disorders limit the ability to function independently, leading to disability, and come at a great personal and social cost.

After denervation, the muscle tissue gradually atrophies, causing necrosis and fibrosis of the skeletal muscles and, eventually, loss of function. In such situations, any nerve repair and regeneration efforts are the optimal choice to regain the lost function, and if the muscle atrophy is slight, the lost function can be restored during reinnervation. Accelerating nerve regeneration and delaying skeletal muscle atrophy are the main treatment strategies to promote the recovery of skeletal muscle function [61]. Massage can regulate the expression of related myogenic factors, promote the proliferation of muscle satellite cells, improve the structure and morphology of skeletal muscles, accelerate the regeneration of muscle fibers, and delay skeletal muscle atrophy. It can have a positive effect on patients’ psyche and emotions. As we highlighted earlier, BDNF secretion is involved in many biochemical and anti-inflammatory processes underlying behavioral, emotional, and cognitive responses, as well as improving mood (antidepressant effects) [16]. Moreover, we conclude that BDNF secretion can promote neuronal communication and nerve regeneration.

## 5. Limitations

In our study, we proved that self-massage with a roller is a technique that stimulates BDNF secretion. To our knowledge, this is one of the first studies of this kind conducted in healthy men, so they should be confirmed on a larger group of subjects, including women, athletes, and patients with various conditions in which massage is an indicated form of therapy. The scope of the study should also be expanded to include analysis of muscle damage markers in athletes after injury to observe whether there is a correlation between the degree of muscle fiber damage, the dynamics of recovery, and BDNF concentrations. In addition, future studies should be carried out in patients with muscle atrophy to analyze whether there is a correlation between IGF-1, other pro-hypertrophic markers, BDNF levels, an increase in muscle circumference, and an improvement in the subjects’ mood or cognitive processes.

## 6. Conclusions

Our research is the first study that confims the influence of foam rollers massage on BDNF secretion. The detailed potential mechanisms presented here could explain many benefits of massage observed in healthy men and in patients with different disorders. However, it ought to be confirmed in different populations. Through this study we wanted to underline that expression/secretion of BDNF during massage could be a strong and potential mechanism of crosstalk between organs and tissues.

## Figures and Tables

**Table 1:** Anthropological characteristics of the study groups.

Parameter	Massage Group (M)		Control Group (C)	
	Before	After	Before	After
Age (yrs)	20.33 ± 0.52		20.67 ± 1.15	
Body height (cm)	178.20 ± 5.19		181.83 ± 5.34	
Body mass (kg)	75.90 ± 6.42	76.25 ± 5.30 *	80.33 ± 7.59	78.67 ± 4.21 *
BMI (kg/m^2^)	24.00 ± 1.39	23.53 ± 1.70 *	24.40 ± 0.95	23.90 ± 0.20 *
FAT (%)	13.67 ± 2.47	14.27 ± 2.32 *	15.33 ± 2.08	13.17 ± 0.99 *
FAT (kg)	10.40 ± 2.29	10.87 ± 1.93 *	12.43 ± 2.95	10.37 ± 1.33 *
FFM (kg)	65.43 ± 5.40	65.38 ± 4.94 *	67.90 ± 4.64	68.30 ± 2.88 *
Muscle mass (kg)	62.17 ± 5.18	62.13 ± 4.71 *	64.53 ± 4.43	64.93 ± 2.76 *
BW (%)	60.85 ± 1.92	60.15 ± 1.55 *	58.57 ± 2.55	60.67 ± 1.74 *
Bone mass (kg)	3.25 ± 0.24	3.25 ± 0.24 *	3.37 ± 0.21	3.37 ± 0.12 *
BMR (kcal/day)	1944.0 ± 164.06	1943.0 ± 148.06 *	2020.67 ± 142.07	2023.67 ± 84.09 *

* ns—in comparison to the value before the experiment and between groups after the experiment.

## Data Availability

The data presented in this study are available on request from the corresponding author.
